# Heterogeneous Presynaptic Distribution of Munc13 Isoforms at Retinal Synapses and Identification of an Unconventional Bipolar Cell Type with Dual Expression of Munc13 Isoforms: A Study Using Munc13-EXFP Knock-in Mice

**DOI:** 10.3390/ijms21217848

**Published:** 2020-10-22

**Authors:** Kaspar Gierke, Julia von Wittgenstein, Maike Hemmerlein, Jenny Atorf, Anneka Joachimsthaler, Jan Kremers, Benjamin H. Cooper, Frederique Varoqueaux, Hanna Regus-Leidig, Johann Helmut Brandstätter

**Affiliations:** 1 Department of Biology, Animal Physiology, Friedrich-Alexander-Universität Erlangen-Nürnberg, 91058 Erlangen, Germany; kaspar.gierke@fau.de (K.G.); julia.wittgenstein@fau.de (J.v.W.); maike.hemmerlein@fau.de (M.H.); hanna.regus-leidig@fau.de (H.R.-L.); 2 Department of Ophthalmology, University Hospital Erlangen, Friedrich-Alexander-Universität Erlangen-Nürnberg, 91054 Erlangen, Germany; jenny.atorf@klinikum-karlsruhe.de (J.A.); anneka.joachimsthaler@uk-erlangen.de (A.J.); jan.kremers@uk-erlangen.de (J.K.); 3 Department of Molecular Neurobiology, Max Planck Institute of Experimental Medicine, 37075 Göttingen, Germany; cooper@em.mpg.de; 4 Department of Computational Biology, University of Lausanne, 1015 Lausanne, Switzerland; Frederique.Varoqueaux@unil.ch

**Keywords:** Munc13-1, ubMunc13-2, brMunc13-2, Munc13-3, ribbon synapse, conventional synapse, retina, type 6 ON bipolar cell

## Abstract

Munc13 isoforms are constituents of the presynaptic compartment of chemical synapses, where they govern important steps in preparing synaptic vesicles for exocytosis. The role of Munc13-1, -2 and -3 is well documented in brain neurons, but less is known about their function and distribution among the neurons of the retina and their conventional and ribbon-type chemical synapses. Here, we examined the retinae of Munc13-1-, -2-, and -3-EXFP knock-in (KI) mice with a combination of immunocytochemistry, physiology, and electron microscopy. We show that knock-in of Munc13-EXFP fusion proteins did not affect overall retinal anatomy or synapse structure, but slightly affected synaptic transmission. By labeling Munc13-EXFP KI retinae with specific antibodies against Munc13-1, -2 and -3, we found that unlike in the brain, most retinal synapses seem to operate with a single Munc13 isoform. A surprising exception to this rule was type 6 ON bipolar cells, which expressed two Munc13 isoforms in their synaptic terminals, ubMunc13-2 and Munc13-3. The results of this study provide an important basis for future studies on the contribution of Munc13 isoforms in visual signal processing in the mammalian retina.

## 1. Introduction

Transgenic mice expressing genetically encoded fluorescent fusion proteins have become a valuable tool in neurobiology research for the analysis of the distribution, dynamics, and interactions of neuron-specific proteins in health and disease. One of their biggest advantages is the intrinsic fluorescence property of green fluorescent protein (GFP) and its derivatives (XFP), allowing the direct visualization of target proteins with non-invasive imaging techniques [1]. The classical method to generate such mouse lines relies on random integration of a promotor-fusion-protein-cassette into the mouse genome, which usually leads to the overexpression of the tagged protein. High expression levels of the tagged proteins are beneficial for their visualization, but can also disturb the biochemical homeostasis of the target cells by interfering with the endogenous gene regulatory mechanisms. This may result in artificial conditions, which are difficult to interpret [2]. To study fluorescently tagged proteins in their native environment under the control of their endogenous regulatory elements, the generation of knock-in (KI) mice in which target proteins are manipulated in their endogenous loci is better suited.

This strategy was successfully employed to generate KI mice for the analysis of Munc13 isoforms [2]. In vertebrates, four Munc13 isoforms (Munc13-1, -2, -3, and -4) are known. Munc13-1, -2, and -3 are specific for neurons and neuroendocrine cells, whereas the more distantly related Munc13-4 is not expressed in the nervous system and plays a role in non-neuronal cells [3,4,5]. Munc13-2 exists as two splice variants, the ubiquitously expressed ubMunc13-2 [6,7] and the brain-specific brMunc13-2 [3,7,8]. In neurons, Munc13 isoforms are critical regulators of vesicle exocytosis [9,10] and the different isoforms shape neurotransmitter release kinetics and short term plasticity in a synapse type-specific manner [11,12,13]. For each of the three neuronally expressed Munc13 isoforms, KI mouse lines were generated by inserting a sequence coding for GFP or YFP at their respective genomic loci. This resulted in the expression of C-terminally tagged fluorescent Munc13-EXFP fusion proteins at endogenous concentrations and locations, i.e., Munc13-1-EYFP, Munc13-2-EYFP and Munc13-3-EGFP, which still underlie all cell type-specific regulatory mechanisms [2,14]. For the Munc13-1-EYFP mouse line, it was shown that the endogenous expression level of the tagged Munc13-1 isoform was sufficient for direct visualization of active zones at brain synapses, and electrophysiological measurements suggested that neurons and synapses in Munc13-1-EYFP mice are capable of normal synaptic transmission [2].

As we have shown previously, all three neuronally expressed Munc13 isoforms are present in the retina [14]. The intricate neuronal network of the retina contains a variety of sensory and higher-order neurons that process in parallel many different aspects of visual signals [15]. Photoreceptor and bipolar cells release the excitatory neurotransmitter glutamate at so-called ribbon synapses to mediate the flow of information through the retina, and horizontal and amacrine cells modulate this information flow by the release of the inhibitory neurotransmitters GABA or glycine at conventional synapses [16,17,18,19]. Interestingly, while Munc13-1 is the dominant Munc13 isoform present in glutamatergic hippocampal synapses [8], glutamatergic retinal ribbon synapses, which are specialized for the fast and sustained release of glutamate [14,20], lack Munc13-1 [21] and express ubMunc13-2 instead [14].

Apart from the unique presence of ubMunc13-2 at retinal ribbon synapses, little is known about the synaptic distribution of the other Munc13 isoforms in the retina. In the present study, we have characterized the retinae of the Munc13-EXFP KI mouse lines. We present a detailed description of the distribution of the Munc13 isoforms in retinal neurons and their chemical synapses, which provides a basis for future studies on the synaptic function of Munc13 isoforms in the mammalian retina.

## 2. Results

### 2.1. Retinal Anatomy, Neuronal Morphology, and Synaptic Ultrastructure Are Normal in Munc13-EXFP KI Mice

**Figure 1:**
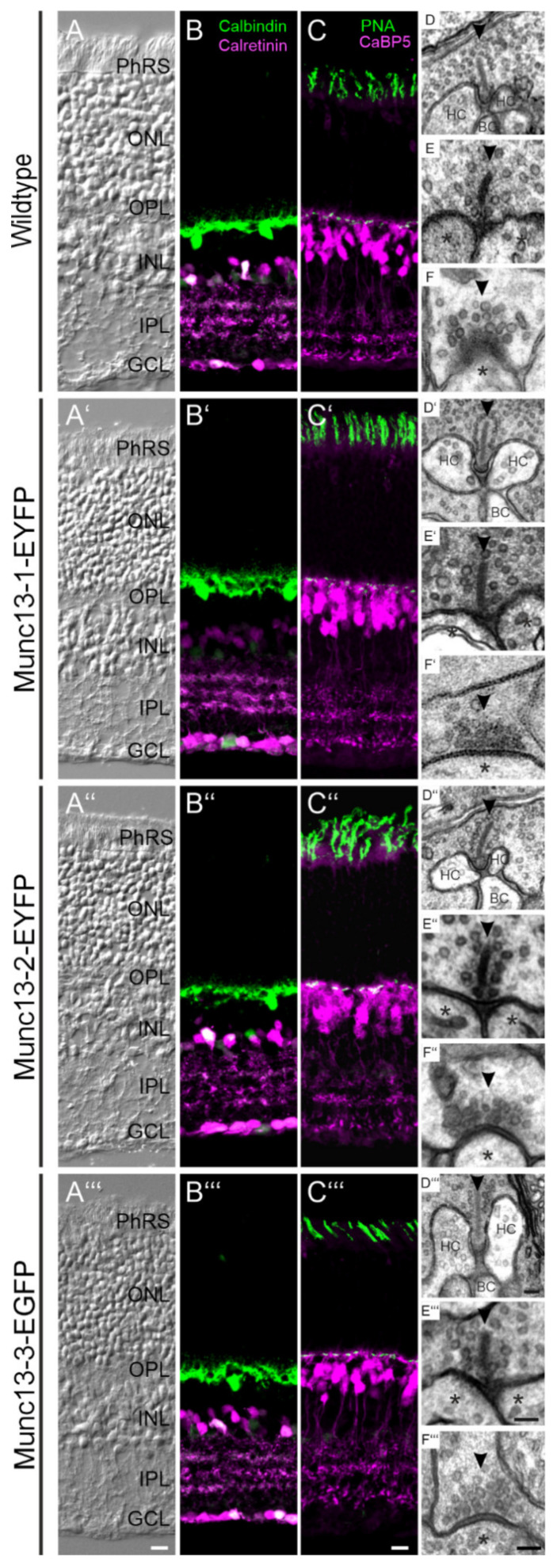
Comparison of retinal anatomy and synaptic morphology between wildtype (WT) and Munc13-EXFP KI mice. (**A**–**A‴**) Nomarski micrographs of vertical cryostat sections through the retinae of WT and Munc13-EXFP KI mouse lines. (**B**–**C‴**) Fluorescence micrographs of vertical cryostat sections through the retinae of WT and the three Munc13-EXFP KI mouse lines double labeled for horizontal cells (anti-Calbindin; green) and amacrine cells (anti-Calretinin; magenta) (**B**–**B‴**) and for cone photoreceptors (PNA; green) and bipolar, amacrine, and ganglion cells (anti CaBP5; magenta) (**C**–**C‴**). (**D**–**F‴**) Electron micrographs of retinal synapses from WT and the three Munc13-EXFP KI mouse lines: rod photoreceptor ribbon synapses (**D**–**D‴**), rod bipolar cell ribbon synapses (**E**–**E‴**), and conventional synapses (**F**–**F‴**). Arrowheads (➤) point to synaptic ribbons (**D**–**E‴**) or active zones (**F**–**F‴**). Asterisks (*) mark postsynaptic processes. PhRS, photoreceptor segments; ONL, outer nuclear layer; OPL, outer plexiform layer; INL, inner nuclear layer; IPL, inner plexiform layer; GCL, ganglion cell layer; HC, horizontal cell; BC, bipolar cell. Scale bar = 20 µm in **A‴** for **A**–**A‴** and in **C‴** for **B**–**C‴** and 0.1 µm in **D‴** for **D**–**D‴**, in **E‴** for **E**–**E‴**, and in **F‴** for **F**–**F‴**.

Until now it has not been examined in detail whether the expression of the Munc13-EXFP fusion proteins in Munc13-1, Munc13-2 and Munc13-3 KI mice affects the overall structure and function of the retina. Thus, we first compared retinal anatomy and neuronal morphology between adult (two months old) wildtype (WT) and Munc13-EXFP KI mice. The comparison of Nomarski micrographs of vertical cryostat sections of WT and KI retinae showed that the Munc13-EXFP fusion proteins did not affect the overall structure of the retina with respect to thickness and layering (Figure 1A–A‴). To visualize the morphology of Munc13-EXFP KI retinae in more detail, we stained vertical retinal cryostat sections with antibodies that label various types of retinal neurons: horizontal cells (anti-calbindin [22]; Figure 1B–B‴), amacrine and ganglion cells (anti-calretinin [23]; Figure 1B–B‴), cone photoreceptors (anti-peanut agglutinin [24]; Figure 1C–C‴), and rod and cone bipolar cells (anti-CaBP5 [25]; Figure 1C–C‴). The various neuron types displayed normal morphology in the Munc13-EXFP KI compared to the WT retinae with respect to their stratification patterns in the two synaptic layers of the retina, the outer (OPL) and inner (IPL) plexiform layer (Figure 1B–C‴). Next, we performed electron microscopy and analyzed qualitatively the ultrastructure of photoreceptor and bipolar cell ribbon synapses and amacrine cell conventional synapses in WT and Munc13-EXFP KI retinae. The expression of the Munc13-EXFP fusion proteins had no obvious effect on pre- and postsynaptic morphology, as illustrated for rod photoreceptor ribbon synapses and their invaginating horizontal and bipolar cell contacts (Figure 1D–D‴), rod bipolar cell ribbon synapses and their amacrine and/or ganglion cell contacts (Figure 1E–E‴), and conventional amacrine cell synapses and their amacrine or ganglion cell contacts (Figure 1F–F‴).

In summary, there was no apparent difference in retinal anatomy, neuronal morphology and synaptic structure between WT and Munc13-EXFP KI mice.

### 2.2. Retinal Function Is slightly Altered in Munc13-EXFP KI Mice

**Figure 2:**
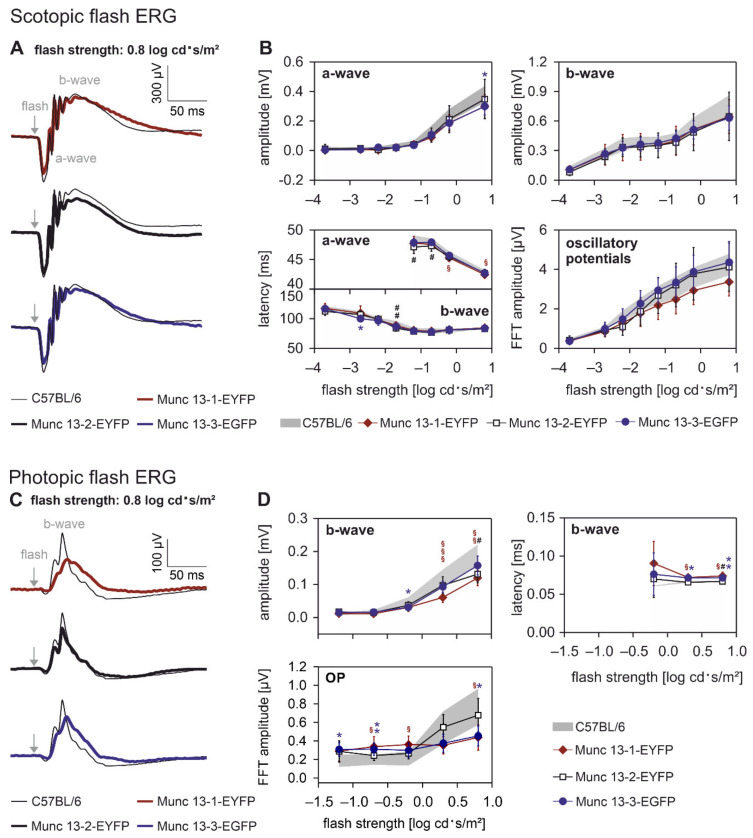
Electroretinographic recordings (ERG) of wildtype (WT) and Munc13-EXFP KI mice. (**A**) Comparison of mean ERG responses to a scotopic flash of 0.8 log cd·s/m^2^ strength between WT (thin black line), Munc13-1-EYFP (red line), Munc13-2-EYFP (thick black line), and Munc13-3-EGFP (blue line) mice. Grey arrows indicate flash onset. Traces show a-wave trough and b-wave peak with oscillatory potentials on its rising part. (**B**) Amplitude and latency of the scotopic a- and b-wave and amplitude of the oscillatory potentials (defined as the maximal amplitude of the Fourier transformed ERG signal between approximately 50 and 100 Hz). WT range is indicated by the gray area. (**C**) Comparison of mean ERG responses to a photopic flash of 0.8 log cd·s/m^2^ strength upon a 1.4 log cd/m^2^ white background measured in WT (thin black line), Munc13-1 (red line), Munc13-2 (thick black line), and Munc13-3 (blue line) mice. Grey arrows indicate flash onset. Traces show only a prominent b-wave peak. (**D**) Averaged amplitude and latency of the photopic b-wave and averaged amplitude of oscillatory potentials as a function of flash strength. *n* = 6 animals per mouse line. All values are presented as mean ± SD. Symbols above lines indicate statistical significances between WT and Munc13-1-EYFP (*), Munc13-2-EYFP (#) or Munc13-3-EGFP (§) mice. Significance levels apply to all of the symbols (*,#,§). * *p* < 0.05; ** *p* < 0.005; *** *p* < 0.001. *p*-values were corrected after Bonferroni for multiple testing.

Next, we examined whether the expression of the Munc13-EXFP fusion proteins altered retinal function. We performed electroretinographic (ERG) recordings under scotopic (dark adapted, rod and combined rod-cone photoreceptor mediated) and photopic (light adapted, cone photoreceptor mediated) conditions and compared the results between WT and the three Munc13-EXFP KI mouse lines (Figure 2). We analyzed the amplitudes and latencies of the a- and b-wave, which originate in photoreceptors and bipolar cells [26] and the oscillatory potentials (OPs), which supposedly represent feedback loops from amacrine to bipolar cells in the inner retina [27].

*Scotopic flash ERGs*. Under scotopic (dark adapted) conditions at a flash strength of 0.8 log cd·s/m^2^, ERG responses of WT retinae displayed a typical response containing an a-wave (first trough), followed by the b-wave peak with OPs on its rising flank (Figure 2A). Under these conditions, all of the Munc13-EXFP KI mice displayed ERG responses similar to their WT controls (Figure 2A). For a detailed comparison of the scotopic flash ERGs between WT and Munc13-EXFP KI mice, we analyzed the amplitudes and latencies of the a- and b-wave as well as the amplitudes of the OPs in the frequency domain (after fast Fourier transformation) at all tested flash strengths (Figure 2B). The amplitudes of a- and b-wave and of the OPs increased with increasing flash strength and there was no significant difference between WT and the three Munc13-EXFP KI mouse lines (Figure 2B). The average latencies of the scotopic a- and b-wave decreased with increasing flash strength, and the Munc13-EXFP KI mice displayed very similar timing properties of the a- and b-wave compared to WT controls (Figure 2B).

*Photopic flash ERGs*. We next measured ERG responses of WT and Munc13-EXFP KI retinae to a photopic (light adapted) flash of 0.8 log cd·s/m^2^ strength upon a rod photoreceptor saturating 1.4 log cd/m^2^ background light. Photopic flash ERGs are typically dominated by the b-wave peak and display only a small a-wave (Figure 2C). Conspicuous are the less pronounced OPs in the Munc13-1 and the Munc13-3-EGFP mice (Figure 2C,D), suggesting that inner retinal activity—possibly the signaling between amacrine and bipolar cells—is impaired in these two mouse strains under photopic conditions. The b-wave amplitudes increased with increasing flash strengths in all three Munc13-EXFP KI mouse lines, but their amplitudes were slightly decreased compared to WT mice (Figure 2D). This was especially evident for Munc13-1-EYFP mice at higher flash strengths (Figure 2D). In addition to the decrease in b-wave amplitudes, the timing of the photopic b-wave was slightly delayed in Munc13-1-EYFP and Munc13-3-EGFP mice, while Munc13-2-EYFP mice showed responses that were highly comparable to WT mice (Figure 2D).

In summary, the expression of the Munc13-EXFP fusion proteins had no influence on the scotopic ERG and mainly affected the photopic ERG components that reflect inner retinal activity.

### 2.3. Analysis of Munc13 Fusion Protein Expression in Munc13-EXFP KI Retinae

**Figure 3:**
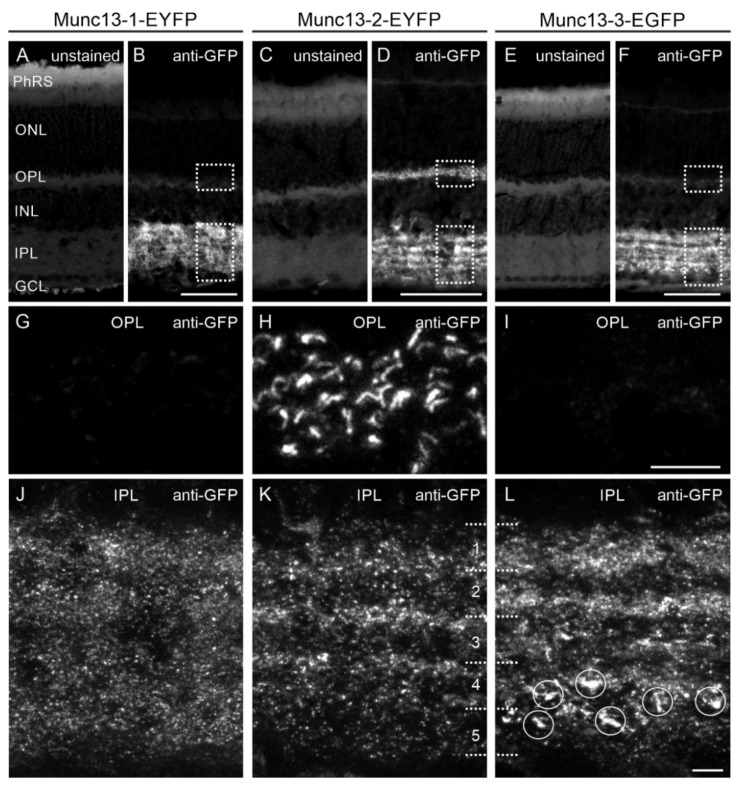
Visualization of Munc13-EXFP fusion proteins in Munc13 KI retinae. (**A**–**F**) Fluorescence micrographs of vertical cryostat sections of unfixed and unstained (**A**,**C**,**E**) and PFA-fixed and anti-GFP antibody-enhanced (**B**,**D**,**F**) retinae of the three Munc13-EXFP mouse lines. (**G**–**L**) High power views of the antibody-enhanced Munc13-EXFP signals in the outer plexiform layer (OPL; (**G**–**I**)) and inner plexiform layer (IPL; (**J**–**L**)). Dotted boxes in (**B**,**D**,**F**) demarcate the respective regions shown in (**G**–**L**). Dashed lines in (**K**,**L**) subdivide the IPL into five strata. Circles in (**L**) highlight large Munc13-3-EGFP clusters. PhRS, photoreceptor segments; ONL, outer nuclear layer; OPL, outer plexiform layer; INL, inner nuclear layer; IPL, inner plexiform layer; GCL, ganglion cell layer. Scale bar = 20 µm in (**B**) for (**A**,**B**), in (**D**) for (**C**,**D**), and in (**F**) for (**E**,**F**). and 5 µm in (**I**) for (**G**–**I**) and in (**L**) for (**J**–**L**).

In vivo imaging assays require bright fluorescence of the reporter signals for sufficient detection. As we found in our previous study that in all three Munc13-EXFP KI retinae the intrinsic fluorescence of the Munc13-EXFP fusion proteins was too weak for direct visualization [14], we re-analyzed this finding by evaluating non-fixed as well as paraformaldehyde (PFA)-fixed retinal tissue. The endogenous fluorescence of the Munc13-EXFP fusion proteins was only detectable as a weak and diffuse signal in the two plexiform layers of the Munc13 KI retinae independent of the tissue processing (Figure 3A,C,E). In addition, photoreceptor segments showed diffuse fluorescence signals, which can be attributed to the intrinsic auto-fluorescence trait of retinal tissue, particularly noticeable after the long exposure times needed to detect the weak Munc13-EXFP signals (Figure 3A,C,E).

In order to enhance the Munc13-EXFP fusion protein signals, we used anti-GFP antibodies on PFA-fixed tissue (Figure 3; [14]). With this approach, Munc13-EXFP signal was intense and restricted to the two plexiform layers of the Munc13-EXFP KI retinae (Figure 3B,D,F,G–L). While Munc13-1-EYFP and Munc13-3-EGFP signals were absent from the OPL (Figure 3G,I), Munc13-2-EYFP signals were discernable as a horseshoe-shaped staining typical for the active zone of photoreceptor synapses (Figure 3H). This is consistent with the sole expression of ubMunc13-2 in photoreceptor ribbon synapses [14]. In the IPL, which we subdivided into five strata according to Cajal [28], punctate Munc13-1-EYFP signals were homogeneously distributed throughout all strata (Figure 3J), while the punctate Munc13-2-EYFP and Munc13-3-EGFP signals were detectable in distinct strata (Figure 3K,L). In addition, Munc13-3-EGFP labeling was visible in larger immunoreactive clusters positioned in the innermost strata 4/5 of the IPL close to the ganglion cell layer (Figure 3L; circles), which is in line with a previously reported finding [14].

### 2.4. The Munc13-EXFP Fusion Protein Signals Match the Munc13 Isoform-Specific Antibody Signals

**Figure 4:**
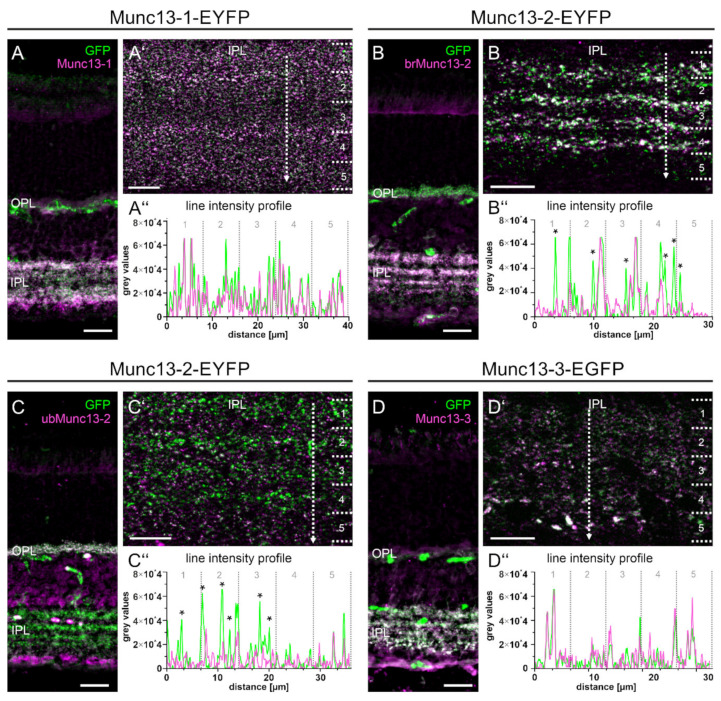
Munc13-EXFP fusion proteins match Munc13 antibody labelings. (**A**–**D**) Fluorescence micrographs of vertical cryostat sections through retinae of Munc13-EXFP mouse lines, double labeled with antibodies against GFP and the respective Munc13 isoform. (**A’**–**D’**) High power views of the inner plexiform layer (IPL). Dotted lines indicate the location of extracted line intensity profiles. Numbers 1–5 indicate the five strata of the IPL. (**A’’**–**D’’**) Analysis of the fluorescence line intensity profiles. Asterisks (*) in (**B’**,**C’**) highlight non-overlapping EXFP-/Munc13 antibody-positive signals. OPL, outer plexiform layer. Scale bar = 20 µm in (**A**–**D**) and 10 µm in (**A’**–**D’**).

To ensure that the Munc13-EXFP fusion proteins represent their respective Munc13 isoforms, we next double labeled Munc13-1-, -2-, and -3-EXFP KI retinae with antibodies against GFP and the corresponding isoform-specific Munc13 antibodies (Figure 4). To visualize the overlap between Munc13-EXFP signals and Munc13 antibody labeling in the IPL, we used fluorescence line intensity profiles positioned in strata 1–5 of the IPL. In Munc13-1-EYFP KI retinae, co-labeling with the Munc13-1-specific antibody resulted in an almost complete overlap of the two fluorescence signals in all five strata of the IPL (Figure 4A–A’’). In Munc13-2-EYFP KI retinae, the distribution pattern of the EYFP signal represents both Munc13-2 isoforms, brMunc13-2 and ubMunc13-2 [14]. This is clearly visible in the OPL, with the absence of brMunc13-2 and the presence of ubMunc13-2 immunoreactivity (Figure 4B,C), and in the IPL with the differential overlap of brMunc13-2 and ubMunc13-2 immunoreactive puncta with Munc13-2-EYFP signals (Figure 4B’,C’). In Munc13-3-EGFP retinae, the GFP signal and the Munc13-3 antibody labeling almost completely overlapped throughout strata 1–5 of the IPL (Figure 4D–D’’).

In summary, the Munc13-EXFP fusion proteins faithfully match the distribution of the respective native Munc13 isoforms.

### 2.5. The Majority of Retinal Synapses Contains Only One Munc13 Isoform

**Figure 5:**
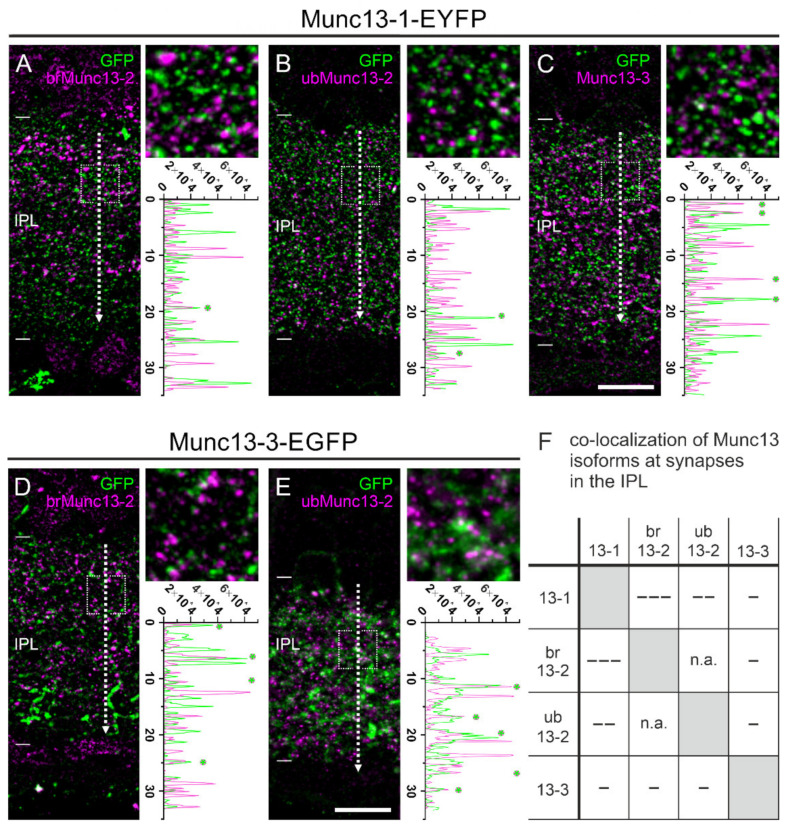
Distribution of Munc13-1, ubMunc13-2, brMunc13-2, and Munc13-3 at synapses in the inner plexiform layer (IPL) of mouse retina. (**A**–**C**) Fluorescence micrographs of the IPL of Munc13-1-EYFP KI retinae double labeled with antibodies against GFP/ubMunc13-2 (**A**), GFP/brMunc13-2 (**B**), and GFP/Munc13-3 (**C**). (**D**–**E**) Fluorescence micrographs of the IPL of Munc13-3-EGFP KI retinae double labeled for GFP/ubMunc13-2 and GFP/brMunc13-2 (**E**). Dotted lines in (**A**–**E**) indicate the location of extracted line intensity profiles. Dotted boxes in (**A**–**E**) demarcate the regions shown in the respective higher power views. (**F**) Table summarizing the degree of co-localization between the different retinal Munc13 isoforms. [- - -] lack of co-localization, [- -] rare co-localization, [-] occasional co-localization. n.a. = not analyzed. Asterisks (*) in line intensity profiles indicate overlap between fluorescence signals. Scale bar = 10 µm in C for (**A**–**E**).

In the brain, most neurons express Munc13-1 and at least one of the other Munc13 isoforms at their synapses [8,13]. For the retina, it is known that photoreceptor ribbon synapses contain only ubMunc13-2 [14], but otherwise little is known about the presence of the Munc13 isoforms in the various types of retinal neurons and their synapses. Because the anti-Munc13 antibodies used in our study were all raised in the same species [14,29], we analyzed the presence of Munc13 isoforms at retinal synapses by combining anti-GFP labeling of the Munc13-EXFP KI retinae with antibody labeling against brMunc13-2, ubMunc13-2, and Munc13-3 (Figure 5).

Munc13-1-EYFP signals, which were discernable throughout the IPL as discrete puncta, co-localized only very rarely with brMunc13-2, ubMunc13-2 or Munc13-3 as seen by the absence of overlapping peaks in the line intensity profile (Figure 5A–C,F). Similarly, we labeled Munc13-3-EGFP retinae with antibodies against brMunc13-2 and ubMunc13-2 (Figure 5D,E). We neither observed considerable signal overlap between Munc13-3-EGFP and brMunc13-2 signals (Figure 5D,F), nor for Munc13-3-EGFP and ubMunc13-2 signals (Figure 5E,F). The combination of the two Munc13-2 splice variants ubMunc13-2 and brMunc13-2 was not tested for co-localization, as the two isoforms are indistinguishable in the Munc13-2-EYFP KI retina.

In summary, we observed very few overlapping signals between the different Munc13 isoforms in the IPL. Thus, we conclude that, unlike in the brain, the majority of ribbon and conventional synapses in the retina contain only one specific Munc13 isoform (Figure 5F).

### 2.6. Type 6 ON Bipolar Cells Contain Munc13-3 in Addition to ubMunc13-2

**Figure 6:**
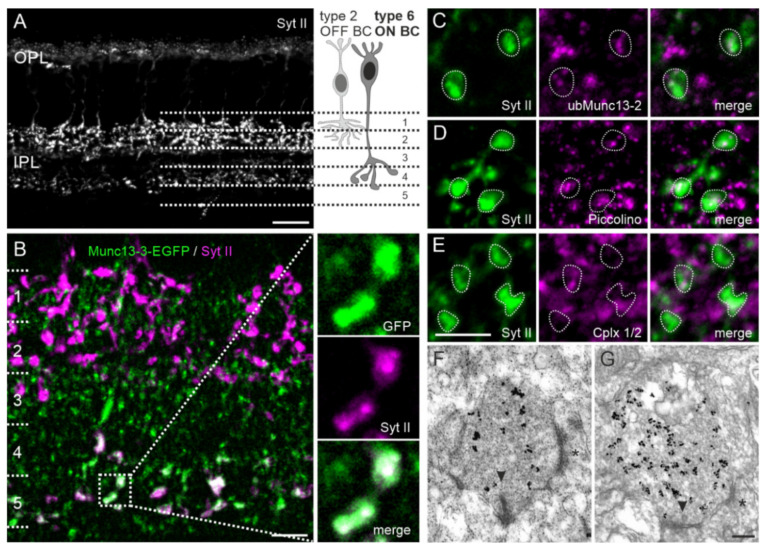
Type 6 ON bipolar cells express Munc13-3. (**A**) Fluorescence micrograph of a vertical cryostat section through wildtype retina labeled with an antibody against Synaptotagmin II (Syt II). Type 2 OFF bipolar cells (BC) are strongly stained in strata 1/2, and type 6 ON BCs are weakly labeled in strata 4/5 of the inner plexiform layer (IPL). (**B**) IPL of a Munc13-3-EGFP KI retina double labeled for GFP (green) and Syt II (magenta) showing co-localization of the large Munc13-3-EGFP positive clusters with Syt II in strata 4/5. Dotted box demarcates the region of the IPL shown in the high power views. (**C**–**E**) High power view of the type 6 ON bipolar cell terminals in strata 4/5 double labeled with antibodies against ubMunc13-2 (**C**), Piccolino (**D**), and Cplx 1/2 (**E**). (**F**,**G**) Representative electron micrographs of preembedding immunolabeled Syt II positive terminals of type 6 ON BCs close to the ganglion cell layer. Terminals of Syt II positive type 6 ON BCs harbor ribbon-containing ((**F**); arrowhead (➤)) and ribbon-free ((**G**); arrowhead (➤)) synaptic contacts. Asterisks (*) in (**F**,**G**) label reciprocal amacrine cell synapses. OPL, outer plexiform layer; Cplx1/2, complexin 1/2. Scale bar = 20 µm in (**A**), 10 µm in (**B**), 5 µm in (**E**) for (**C**–**E**), and 0.2 µm in (**G**) for (**F**,**G**).

Next, we got particularly interested in the large Munc13-3-EGFP-positive clusters in strata 3 and 4 of the IPL, as they clearly differed in appearance from the other Munc13-EXFP signals (Figure 3). To clarify which cell type of the retina contains the Munc13-3-EGFP signal, we examined its co-localization with markers for rod and cone bipolar cells and amacrine and ganglion cells (data not shown). We found a co-localization of the large Munc13-3-EGFP-positive clusters with Synaptotagmin 2 (Syt II) (Figure 6B,C), a synaptic vesicle-associated membrane protein acting as a calcium sensor for fast neurotransmitter release [30]. In mouse retina, antibodies against Syt II mark type 2 OFF bipolar cells, which stratify with their axon terminals in strata 1 and 2 of the IPL [31] and type 6 ON bipolar cells, which stratify in strata 3, 4, and 5 of the IPL [32] (Figure 6A,B). As the expression of Munc13-3 by a ribbon synapse forming neuron was surprising, we further analyzed the type 6 ON bipolar cell for the expression of presynaptic proteins typically present in (i) retinal ribbon synapses, for example, ubMunc13-2 and Piccolino [14,33,34] and (ii) conventional chemical synapses, for example, Cplx1/2 [35]. The type 6 ON bipolar cell terminals contained only ribbon synapse specific proteins apart from Munc13-3 (Figure 6C–E).

Because of the presence of both Munc13-3 (conventional synapse) and ubMunc13-2 (ribbon synapse) in type 6 ON bipolar cell terminals, we next labeled type 6 ON bipolar cells with the anti-Syt II antibody and visualized the ultrastructure of the synaptic sites. We found in the synaptic terminals of type 6 ON bipolar cells both ribbon-containing synaptic sites as well as ribbon-free presynaptic densities that may represent conventional synaptic contact sites (Figure 6F,G), a finding that is in accordance with published data [36,37].

**Figure 7:**
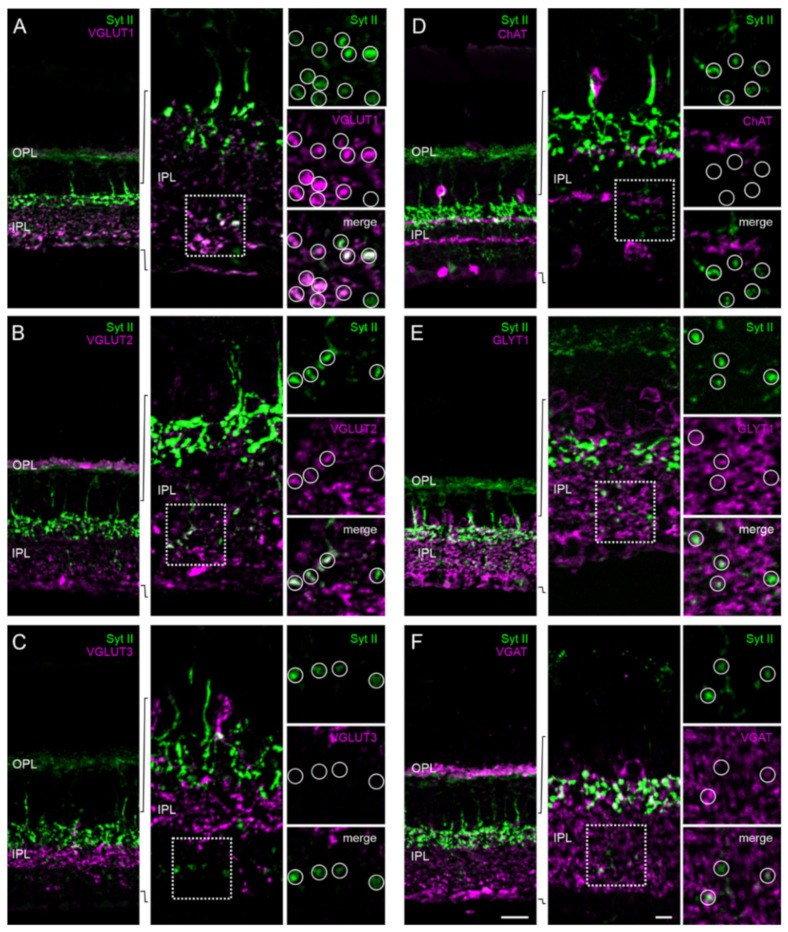
Neurotransmitter phenotype of type 6 ON bipolar cells (BCs). (**A**–**F**) Vertical cryostat sections through wildtype mouse retinae double labeled with an antibody against Syt II to label type 6 ON BCs in combination with markers for glutamatergic synapses VGLUT1 (**A**), VGLUT2 (**B**), and VGLUT3 (**C**), cholinergic synapses ChAT (**D**), glycinergic synapses GLYT1 (**E**), and GABAergic synapses VGAT (**F**). Dotted boxes demarcate the regions in the IPL shown in the high power views. Circles highlight synaptic terminals of type 6 BCs together with the respective synapse markers. OPL, outer plexiform layer; IPL, inner plexiform layer; VGLUT, vesicular glutamate transporter; ChAT, choline acetyltransferase; GLYT1, glycine transporter1; VGAT, vesicular GABA transporter. Scale bars = 20 µm in (**F**) for (**A**–**F**) and 5 µm for the higher power views of the IPL.

The presence of conventional and ribbon synaptic sites and of Munc13-3 and ubMunc13-2 in terminals of type 6 ON bipolar cells raises the intriguing possibility of a combinatory release of different types of neurotransmitters. To pursue this possibility, we labeled type 6 ON bipolar cells in combination with representative markers for glutamatergic synapses (vesicular glutamate transporters 1, 2, and 3 (VGLUT; [38,39,40]), cholinergic synapses (choline acetyltransferase (ChAT; [41,42]), and GABA-/glycinergic synapses (VGAT [43,44] and GLYT1 [45,46]). Syt II-positive terminals of type 6 ON bipolar cells strongly co-localized with VGLUT1 staining (Figure 7A). There was also some co-localization of type 6 ON bipolar cell terminals with VGLUT2 (Figure 7B), but not with VGLUT3 (Figure 7C). The antibody against ChAT stained two distinct strata in the IPL, which did not overlap with the Syt II-positive type 6 ON bipolar cell terminals (Figure 7D). Despite strong GLYT1 labeling throughout the IPL, which sometimes seemed to engulf the type 6 ON bipolar cell terminals, there was no obvious co-localization between Syt II and GLYT1 staining (Figure 7E). Similarly, VGAT signals were often found in close proximity to type 6 ON bipolar cell terminals but did not co-localize with Syt II signals (Figure 7F).

Taken together, type 6 ON bipolar cells are unconventional with regard to the expressed Munc13 isoforms and the presence of ribbon and conventional synaptic sites, but seem to release only one neurotransmitter, which is glutamate.

## 3. Discussion

Synaptic vesicle docking and priming are the last two steps that precede SNARE-mediated exocytosis of synaptic vesicles [47,48]. A dense protein network located in close vicinity to the presynaptic membrane, called cytomatrix at the active zone, tightly regulates these steps [5,49]. Munc13 isoforms are constituents of the cytomatrix at the active zone [50] and are of paramount importance in the priming process of synaptic vesicles [9]. Molecularly, Munc13 isoforms achieve their function by interacting with several components of the active zone and with the SNARE-complex [7,51,52,53]. In line with the central role of Munc13 isoforms in neurotransmitter release, removal of Munc13-1 from brain synapses leads to the almost complete cessation of neurotransmitter release from glutamatergic synapses [9]. Interestingly, glutamatergic synapses of the retina, the ribbon synapses of photoreceptor and bipolar cells, which stratify with their synaptic terminals in the OPL and IPL, respectively, do not contain Munc13-1 [21].

In a previous study on Munc13 isoforms in the retina, we have used a Munc13-1-EYFP KI mouse line generated by Kalla and colleagues [2] and have shown that glutamatergic retinal ribbon synapses express ubMunc13-2, a splice variant of Munc13-2, instead of Munc13-1 [14]. Genetic deletion of ubMunc13-2 in a Munc13-2 knock-out mouse had little effect on photoreceptor ribbon synaptic transmission, suggesting that synaptic vesicle priming differs fundamentally between ribbon and conventional synapses [14]. This assumption is supported by findings on inner hair cells in the cochlea, which also form ribbon synapses, but operate independently of Munc13 isoforms altogether [54].

### 3.1. Munc13-EXFP KI Mouse Lines, a Tool for In Vivo and In Vitro Retinal Studies?

In the present study, we have expanded our previous findings by performing an in-depth analysis of the retinae of the three Munc13-EXFP KI mouse lines Munc13-1-EYFP, Munc13-2-EYFP, and Munc13-3-EGFP [2]. We investigated retinal morphology, retinal physiology and distribution patterns of the individual Munc13 isoforms. Morphologically, the Munc13-EXFP fusion proteins compromised neither gross retinal anatomy, nor neuronal morphology or synaptic ultrastructure (Figure 1). As assessed by electroretinographic recordings, the addition of the EXFP tag did not significantly alter retinal responses under conditions of low light intensities (Figure 2; scotopic condition). Under conditions of high light intensities, however, especially Munc13-1-EYFP retinae showed a decrease in b-wave amplitude at higher flash intensities and a delay in the timing of the b-wave compared to WT controls (Figure 2; photopic condition). This is surprising based on the expression of Munc13-1 at conventional amacrine cell synapses and its absence from photoreceptor ribbon synapses, and we are not aware of any case where amacrine cell signaling affects b-wave characteristics. Furthermore, the OPs were decreased in Munc13-1-EYFP and Munc13-3-EGFP mice under photopic luminance conditions, suggesting altered signaling in the inner retina. OPs are thought to reflect feedback loops between amacrine and bipolar cells [27], hence decreased OPs might correlate with altered neurotransmitter release in both cell types. This indicates that the addition of the EYFP tag to Munc13-1 slightly perturbs the function of Munc13-1 containing retinal synapses, contrary to Munc13-1-EYFP containing brain synapses, which were functionally identical to those of WT controls [2].

In our previous study, we also analyzed the usefulness of Munc13-EXFP KI mouse lines for imaging studies on the retina [14]. Unlike in the brain, where the Munc13-1-EYFP fluorescent synapses are readily amenable for imaging [2], the intrinsic fluorescent signal of the Munc13-EXFP fusion proteins under their native promotor was very weak in the retina and immunostaining of the EXFP tag had to be performed for visualization [14]. As formaldehyde fixation is known to considerably quench XFP fluorescence [55], we reinvestigated whether omitting fixation would yield brighter signals. Unfortunately, EXFP signals were still indistinguishable from background fluorescence (Figure 3). Thus, we conclude that Munc13 KI mice are not suitable for in vitro functional studies of the retina, for example, for ex-vivo slice preparations for patch clamp recordings. Nevertheless, we confirm that the Munc13-EXFP fusion protein signals faithfully match the distribution patterns and expression levels of the respective Munc13 isoforms (Figure 4), showing that the Munc13-EYFP KI mouse lines are useful tools to study synaptic distribution of Munc13 isoforms in the retina (Figure 5, Figure 6 and Figure 7).

### 3.2. Mutually Exclusive Presence of Munc13 Isoforms at Retinal Synapses

The distribution of Munc13-1 throughout the IPL (Figure 3 and Figure 4) was in accordance with the ubiquitous expression of Munc13-1 in brain neurons [8]. Despite the wide expression of Munc13-1 in the IPL, we show that Munc13-1 rarely co-localized with Munc13-2 or Munc13-3 (Figure 5), indicating that subsets of retinal synapses in the IPL operate independently of Munc13-1. Most likely, many of these synapses correspond to ribbon synapses of bipolar cells, which stratify with their synaptic terminals in the different strata of the IPL and have been shown to contain ubMunc13-2 [14,21]. Our data further indicate that a large proportion of conventional synapses in the retina operates with only one Munc13 isoform because also brMunc13-2 and Munc13-3 rarely co-localized with other Munc13 isoforms (Figure 5). This is in contrast to many brain synapses as, for example, synapses in the hippocampus, in the cerebellum and in the calyx of held rely on more than one Munc13 isoform [10,11,13,56].

Taken together, our results indicate that ribbon as well as conventional synapses of the retina each operate primarily with a single Munc13 isoform. This is particularly interesting in view of the fact that retinal function depends on a huge heterogeneity of neurons. Amacrine cells, of which more than sixty different types are known [57], contact other amacrine cells, bipolar cells, and ganglion cells in the IPL with conventional synapses. They comprise the functionally most heterogeneous class of retinal neurons [58,59] and the presence of given Munc13 isoforms at their synapses might contribute an additional layer of specificity to ultimately shape the response of ganglion cells to visual signals and thus the output of the retina to the brain [57].

### 3.3. Dual Expression of Munc13 Isoforms in Synaptic Terminals of Type 6 ON Bipolar Cells

In view of the selective expression of Munc13 isoforms at given retinal synapses, we assumed that ubMunc13-2 represents the “ribbon-specific” Munc13 of photoreceptor and bipolar cell ribbon synapses, while Munc13-1, brMunc13-2, and Munc13-3 represent the “conventional” Munc13 isoforms of amacrine cell synapses ([14]; this study). Yet we were intrigued to find on few occasions a signal overlap of Munc13-3 with the other Munc13 isoforms (Figure 5), in particular regarding its expression at synaptic terminals of type 6 ON bipolar cells, known to express ubMunc13-2 (Figure 6). This apparent co-expression would be in accordance with the discussed role of Munc13-3 as a modulator of Munc13-1 and Munc13-2 [13,56,60]. Yet we show with electron microscopy that type 6 ON bipolar cells form both ribbon-containing and ribbon-free synaptic sites (see also [36,37]). Hence, the dual expression of Munc13 isoforms may not be functionally promiscuous, but correspond to specific synaptic sites in type 6 ON bipolar cells terminals. Neurotransmitter release at ribbon-containing (with ubMunc13-2) or ribbon-free (with Munc13-3) synaptic sites of type 6 ON bipolar cells could be governed by different presynaptic molecular machineries, yielding different release properties. Such a phenomenon has recently been demonstrated for certain types of amacrine cells in the retina, able to release different neurotransmitters at neighboring release sites. VGLUT3-positive amacrine cells, for example, are able to release the canonical inhibitory amacrine cell neurotransmitter glycine and the excitatory neurotransmitter glutamate from different synaptic sites [22,61,62,63,64]. For a VGLUT1-positive amacrine cell, it was only recently shown that it could potentially also mediate dual excitatory glutamatergic and inhibitory GABAergic transmission [57]. The results of our experiments, in which we examined the presence of the major retinal neurotransmitters glutamate, acetylcholine, glycine, and GABA in synaptic terminals of type 6 ON bipolar cells, however, did not provide evidence for the presence of a neurotransmitter other than glutamate (Figure 7).

Another interesting possibility could be that dependent on the presence of ubMunc13-2 and Munc13-3 at ribbon-containing and ribbon-free synaptic sites, type 6 ON bipolar cells release glutamate with different release properties, for example, sustained versus transient. By this, they could differentially regulate the activity of their postsynaptic target neurons. For excitatory hippocampal neurons, it was shown in autaptic cell cultures that they express both Munc13-1 and Munc13-2 and that the two Munc13 isoforms differentially control vesicle priming and synaptic transmission. While Munc13-1-dependent hippocampal synapses depressed upon repeated stimulation, Munc13-2-dependent synapses showed synaptic facilitation [11].

In conclusion, the finding that ribbon and conventional synapses operate preferentially with single Munc13 isoforms, and that solely type 6 ON bipolar cells terminals express both ubMunc13-2 and Munc13-3 isoforms, further specifies the intricate specificity and heterogeneity of presynaptic machineries, which contribute to the complexity of visual signal processing in the mammalian retina.

## 4. Materials and Methods

### 4.1. Animals

Animal experiments were approved by the local authorities (Regierung von Mittelfranken, AZ 55.2.2-2532.2-825-18; Amt für Veterinärwesen der Stadt Erlangen, AZ TS10/07) and conducted in accordance with the European Communities Council Directive (2010/63/EU). Munc13-EXFP KI mice used in the present study were generated and described by Kalla and colleagues [2,14]. Briefly, the genes of the three Munc13 isoforms (Munc13-1, -2 and -3) were modified within their genomic loci by homologous recombination. Each strain expresses one of the fluorescently tagged Munc13 fusion proteins, which still underlie all regulatory mechanisms of the cell: Munc13-1-EYFP, Munc13-2-EYFP or Munc13-3-EGFP. Munc13-EXFP KI mice and C57BL/6 mice were group housed at the animal care facility of the FAU Erlangen-Nürnberg under a 12-h light/dark cycle with ad libitum access to food and water. If not stated otherwise, three different animals per genotype (WT, Munc13-1-EYFP, Munc13-2-EYFP, and Munc13-3-EGFP) were used for each experiment.

### 4.2. Antibodies

The following primary antibodies were used for immunocytochemistry (ICC) and immuno electron microscopy (EM): rabbit anti-CaBP5 (ICC 1:500; [25]), rabbit anti-Calbindin D28k (ICC 1:1000; Swant, Marly, Switzerland, cat. no. CB-38), mouse anti-Calretinin (ICC 1:2000; Chemicon, Temecula, CA, USA, cat. no. MAB1568), guinea pig anti-Complexin 1/2 (ICC: 1:500; Synaptic Systems GmbH, Göttingen, Germany, cat. no. 122 002); mouse anti-GFP (ICC 1:400; Chemicon, cat. no. MAB3580), rabbit anti-GFP (ICC 1:2000; Thermo Fisher Scientific, Waltham, MA, USA, cat. no. A11122), rabbit anti-Munc13-1 (batch 40-41; ICC 1:6000; [14]), rabbit anti-ubMunc13-2 (batch 52; ICC 1:6000; [14]), rabbit anti-brMunc13-2 (batch 50; ICC 1:6000; [14]), rabbit anti-Munc13-3 (batch 48; ICC 1:6000; [14]), monoclonal anti-Synaptotagmin II (Syt II or znp-1; ICC 1:2000, EM 1:2000; ZIRC, Eugene, OR, USA, cat. no. ZDB-ATB-081002-25), guinea pig anti-VGLUT1 (ICC 1:25,000, Chemicon, cat. no. AB 5905), rabbit anti-VGLUT2 (ICC 1:1000, Synaptic Systems GmbH), guinea pig anti-VGLUT3 (ICC, 1:3000, Synaptic Systems GmbH, cat. no. 135 204), guinea pig anti-VGAT (ICC 1:5000, Synaptic Systems GmbH, cat. no. 135 403), goat anti-ChAT (ICC 1:400, Merck Millipore, Burlington, MA, USA, cat. no. AB144P), goat anti-GLYT1 (ICC, 1:10,000, Chemicon, cat. no. AB 1770). Primary antibodies were visualized with suitable fluorophore-coupled (ICC) or Nanogold^®^-coupled (EM) secondary antibodies: Alexa^®^ 488/555-conjugated goat anti-mouse and goat anti-rabbit IgG (1:250–1:500; Thermo Fisher Scientific, cat. nos. A11034, A21428), Cy3/Cy5-conjugated goat anti-mouse and goat anti-rabbit IgG (1:100–1:200; Dianova, Hamburg, Germany cat. nos. 115-175-003, 111-165-003), Alexa^®^ 647 donkey anti-goat IgG (1:250–1:500; Thermo Fisher Scientific, cat. no. A-21447), Alexa^®^ 555-conjugated donkey anti-mouse (1:250, Thermo Fisher Scientific, cat. no. A-31570), FluoroNanogold^®^ (FNG; Fab’ Fragments, 1.4 nm diameter)-conjugated goat anti-mouse IgG (EM 1:400; Nanoprobes, Yaphank, NY, USA, cat. no. #2002-0.5ML). Outer segments and synapses of cone photoreceptors were labeled with fluorescein-coupled Peanut Agglutinin (PNA, ICC 1:500, Vector Laboratories, Burlingame, CA, USA, cat. no. FL-1071-10).

### 4.3. Immunofluorescence Staining and Light Microscopy

For light microscopic analysis, retinae of male and female Munc13 KI mice and C57Bl/6 mice were prepared as described previously [65]. Mice were anesthetized deeply with Isofluorane (Abbott Laboratories, Chicago, IL, USA) and killed by cervical dislocation. For the analysis of unfixed tissue, eyecups were directly immersed in Tissue-Tek O.C.T. freezing medium (Sakura Finetek Germany, Staufen, Germany) and immediately frozen using liquid nitrogen cooled isopentane. For immunocytochemistry of fixed tissue, cornea, lens and vitreous body were removed from the eyecup before immersion fixation for 15–30 min in 4% paraformaldehyde (PFA) in phosphate buffer (PB, 0.1 mol L^−1^, pH 7.4). After washing in 0.01 mol L^−1^ phosphate-buffered saline (PBS), retinae were dissected from the eyecups followed by incubation in 10%, 20%, and 30% (*w*/*v*) sucrose in 0.01 mol L^−1^ PBS for cryoprotection. Retinae were mounted in Tissue-Tek O.C.T. freezing medium (Sakura) and cut vertically into 14 µm thick sections using a cryostat (CM3050 S, Leica Microsystems, Wetzlar, Germany). For immunolabeling experiments, frozen cryostat sections were thawed, washed in 0.01 mol L^−1^ PBS and incubated in blocking solution (10% normal goat serum (NGS), 1% bovine serum albumin (BSA), 0.5% Triton-X-100 in 0.01 mol L^−1^ PBS) for 1 h. The blocking solution was replaced by the primary antibody diluted in antibody solution (3% NGS, 1% BSA and 0.5% Triton-X-100 in 0.01 mol L^−1^ PBS) and slices were incubated in a humidified chamber overnight. For experiments using antibodies raised in goat, NGS was replaced by equal amounts of donkey serum. PBS washed sections were incubated with secondary antibodies for 1 h at RT before mounting in Aqua Polymount (Polysciences, Warrington, PA, USA). Labeled sections were analyzed with a Zeiss Axio Imager Z1 equipped with an ApoTome or with an LSM710 (both Carl Zeiss AG, Oberkochen, Germany). Images were acquired with the ZEN blue software (Carl Zeiss) using a 20 × (0.8 NA, Apochromat), a 63 × (1.4 NA oil immersion, Plan Apochromat), or a 100 × (1.3 NA oil immersion, Plan Neofluar) objective as z-stacks of multiple optical sections. Arrangement of images was performed using CorelDRAW X11 (Corel Corporation, Ottawa, ON, Canada).

### 4.4. Best Fix Electron Microscopy

Retinae were prepared for electron microscopy as described previously [66]. For conventional electron microscopy, retinae were fixed in 4% PFA and 2.5% glutaraldehyde for 2 h at room temperature, followed by incubation in 2% osmiumtetroxide (OsO_4_) for 1.5 h. After dehydration in rising EtOH series (30–100%) and propylenoxide retinae were embedded in Epon resin. Ultrathin sections ~55 nm were cut with an Ultracut E microtome (Reichert-Jung/ Leica Biosystems, Nußloch, Germany). Finally, samples were counterstained with lead citrate and uranyl acetate in an automatic contrasting system (EM AC20, Leica Microsystems, Wetzlar, Germany). Ultrathin sections were examined and photographed with an EM10 electron microscope (Carl Zeiss) equipped with a SC1000 Orius^TM^ CCD camera (GATAN, Inc., Pleasanton, CA, USA) in combination with the DigitalMicrograph 3.1 software (GATAN).

### 4.5. Preembedding Immunoelectron Microscopy

For preembedding immunoelectron microscopy of Syt II-positive cells, retinae of C56BL/6J mice were prefixed for 45 min in 4% PFA and 0.02% Picric acid in 0.1 mol L^−1^ PB (pH 7.4) buffer at RT. To improve tissue quality of the IPL, we employed a secondary prefixation step for 18 h in 4% PFA in PB (4 °C, pH 10.4; adapted from [67]). After extensive washing in PBS, retinae were treated with freshly prepared 1% NaBH_4_ in H_2_O followed by rising sucrose solutions (10–30%) for cryoprotection. To further enhance antibody penetration, retinae were subjected to three freeze and thaw cycles in liquid nitrogen before embedding in 3% low melting agarose. Agarose blocks were cut into 70 µm thick sections with a vibratome (VT100S, Leica Microsystems) and blocked in blocking solution before antibody incubation (Syt II, 1:2000 ZIRC; in antibody dilution solution) for 48 h at 4 °C. Secondary antibody labeling was performed using FluoroNanogold^®^-coupled anti mouse antibody (1:400; Nanoprobes) for 3 h at RT. Slices were postfixed in 2.5% glutaraldehyde in 0.1 mol L^−1^ PB. Nanogold particles were enhanced using HQ Silver Kit (Nanoprobes) for 6 min in darkness. Enhancement was stopped by several short washes in 2% sodium acetate (pH 5.5). To render silver-enhanced particles impervious to degradation by OsO_4_ treatment, sections were subjected to gold toning using 0.05% chloroauric acid for 8 min at 4 °C. Gold toning was stopped by brief washing in 2% sodium acetate (pH 5.5) and stabilized in 1% thiosulfate in H_2_O. After several washing steps in 0.1 M cacodylate buffer, sections were subjected to osmication (0.25% OsO_4_ in cacodylate buffer, 30 min, 4 °C). Dehydration and embedding in epoxy resin was performed as described for best fix electron microscopy.

### 4.6. Electroretinographic (ERG) Recordings

ERGs were measured from 6 animals per group (WT, Munc13-1-EYFP, Munc13-2-EYFP, and Munc13-3-EGFP) at the age of 2 months. Measurements from Munc13 KI mice were compared to measurements from C57BL/6 control mice (WT). A detailed description of the experimental procedure and the signal analysis can be found elsewhere [68,69]. Briefly, the animals were dark adapted overnight and all further handling was performed under deep red illumination. Mice were anesthetized by an intramuscular injection of 50 mg/kg ketamine (Ketavet^®^, Pfizer, Berlin, Germany) and 10 mg/kg xylazine (Rompun^®^ 2%, Bayer, Leverkusen, Germany). A subcutaneous injection of saline solution (10 mL/kg, 0.9%) protected the mice from desiccation. Pupils were dilated with a drop of tropicamide (Mydriaticum Stulln^®^, 5 mg/mL, Pharma Stulln GmbH, Stulln, Germany) and phenylephrin-hydrochloride (Neosynephrin POS^®^ 5%, Ursapharm, Saarbrücken, Germany). To measure the ERG, the ground needle electrode was placed subcutaneously at the base of the tail, the reference needle electrodes were positioned subcutaneously medially to the ears and the active contact lens electrodes (Mayo Corporation, Nagoya, Japan), internally covered with Corneregel^®^ (Dr. Mann Pharma, Berlin, Germany), were placed on the cornea of each eye. To deliver the stimuli, a Ganzfeld Bowl (Q450 SC, Roland Consult, Brandenburg, Germany) was used. Stimulation and ERG recording were controlled using the RetiPort system (Roland Consult). The flash strength increased in eight steps (−3.7, −2.7, −2.2, −1.7, −1.2, −0.7, −0.2 and 0.8 log cd·s/m^2^) and, depending on flash strength, 8 to 12 flashes were averaged. Flash duration varied between 5 µs and 5 ms depending on the required total energy. After 5 min adaptation to 1.4 log cd/m^2^ steady background light, photopic flash ERG measurements were performed. Flashes of five strengths (−1.2, −0.7, −0.2, 0.3 and 0.8 log cd·s/m^2^) were superimposed on the background. At each flash strength, 20 responses were averaged. All ERG signals were amplified 100,000 times, band-pass filtered between 1 and 300 Hz, and digitized with a sampling frequency of 2044 Hz. For signal analysis, the amplitudes and implicit times of the a- and b-waves of the flash ERGs were measured after the oscillatory potentials were removed through a variable digital filter procedure [68,69]. The a-wave amplitude was defined from baseline (the average of the 30 ms prestimulus recording) to the a-wave trough. The b-wave amplitude was measured from the trough of the a-wave to the peak of the b-wave. Latencies were measured as time difference between flash onset and time of occurrence of the a-wave trough or the b-wave peak. The OP amplitudes were defined as the maximal amplitude in the frequency domain in the range between 50 and 100 Hz after Fourier transformation of the ERG signal. Statistical analysis was performed using SPSS (IBM, Armonk, NY, USA). All comparisons were performed with unpaired t-tests after testing for Gaussian distribution. *p*-values were corrected after Bonferroni for multiple testing.

### 4.7. Fluorescence Intensity Profile Analysis

For the co-localization analysis of fluorescence signals, images were first subjected to background subtraction using the rolling ball algorithm and were further adjusted for brightness and contrast using ImageJ (NIH, Bethesda, MA, USA). Next, a line was placed in the region of interest and line profiles were generated using the ImageJ “multi plot profile” plugin (BAR repository; [70]). Data were imported into the GraphPad Prism 8.3 software (GraphPad Software Inc., San Diego, CA, USA) for graphical presentation.

## Abbreviations

AC	amacrine cell
BC	bipolar cell
CaBP5	calcium binding protein 5
EXFP	EGFP and its derivative EYFP
GCL	ganglion cell layer
HC	horizontal cell
INL	inner nuclear layer
IPL	inner plexiform layer
KI	knock-in
ONL	outer nuclear layer
OP	oscillatory potentials
OPL	outer plexiform layer
PhRS	photoreceptor segments
PNA	peanut agglutinin
